# *Mori Fructus* Polysaccharides Attenuate Alcohol-Induced Liver Damage by Regulating Fatty Acid Synthesis, Degradation and Glycerophospholipid Metabolism in Mice

**DOI:** 10.3389/fphar.2021.766737

**Published:** 2021-10-22

**Authors:** Liang Bian, Hua-Guo Chen, Xiao-Jian Gong, Chao Zhao, Xin Zhou

**Affiliations:** ^1^ Key Laboratory for Information System of Mountainous Areas and Protection of Ecological Environment, Guizhou Normal University, Guiyang, China; ^2^ Guizhou Engineering Laboratory for Quality Control and Evaluation Technology of Medicine, Guizhou Normal University, Guiyang, China; ^3^ Research Center for Quality Control of Natural Medicine, Guizhou Normal University, Guiyang, China

**Keywords:** nontargeted lipidomics, *Mori fructus* polysaccharide, lipid metabolism, protective effect, hematoxylin and eosin staining

## Abstract

*Mori Fructus* polysaccharides (MFP) are macromolecules extracted from Mori Fructus (MF), which has the biological activity of anti-liver damage. Our group found that MFP maybe down regulate the serum triglyceride level in mice with alcohol-induced liver damage, suggesting that MFP can regulate lipid metabolism, but its specific mechanism is still not clear. Fifty SPF-ICR male mice weighing 18–22 g were randomly divided into five groups, blank group, model group, bifendate group, MFPA1 group and MFPB1 group. The blood and liver tissues were taken from mice for nontargeted lipidomic analysis and histopathological examination after 7 day’s treatment. The histopathological changes indicated that the normal liver cells were intact and regular, with orderly arrangement and distinct cell boundaries; the liver of model mice showed inflammatory infiltration, ballooning degeneration in the cells and small lipid drops; the liver of mice in the bifendate, MFPA1 and MFPB1 groups showed similar symptoms to those of model mice, but the lesions were less severe and the ballooning degeneration were reduced. Multivariate analysis of all lipids in the serum of five groups of mice showed there were obvious differences in lipid metabolism between the model group and the blank group. At the same time, seven kinds of differential lipids were precisely identified after screening, including prostaglandins, long-chain fatty acids, glycerophospholipids, acyl carnitines. In summary, alcohol intake and MFP intervention have significant effects on fatty acid synthesis, degradation and glycerophospholipid metabolism.

## Introduction

Ethanol is metabolized in the liver to produce toxic substances such as acetaldehyde, which increases the intracellular NADH/NAD^+^ ratio, inhibits the β-oxidation of fatty acids, increases the esterification rate of fatty acids and causes the accumulation of triglycerides in the liver, ultimately causing a fatty liver (Ramachandran et al., 2019). World Health Organization released the Global status report on alcohol and health in 2018, which states that approximately 3 million people die from alcohol-induced liver disease each year worldwide, accounting for nearly 5.3% of all deaths (World Health Organization, 2018). The current treatment of alcohol-induced liver disease is mainly drug therapy and surgical operation, but most drug therapy have side effects and high recurrence rate, for example, glucocorticoids and phosphatidylcholine cause disorders of lipid metabolism, while taurine and bile salts place a high burden on the gastrointestinal tract, so the search for a low toxicity and high efficiency drug has become a hot spot in the field of alcohol-induced liver disease.

*Mori Fructus* (*Morus alba L*. fruit, mulberry) is a kind of effective Chinese herbs for the treatment of alcoholic intoxication and has a long history of use. MFP is a kind of low side effect macromolecule which is used to treat alcohol-induced liver disease (Zhou et al., 2017). Many researches indicate that MFP can treat alcohol-induced liver disease, but the mechanism of MFP against alcohol-induced liver disease has not been elucidated. Studies have shown that alcohol could induce lipid peroxidation in the liver, leading to steatosis (Kotronen et al., 2010). Therefore, the present study explores the lipid metabolism mechanism of MFP against acute alcohol-induced liver damage.

Lipidomics can detect the composition and content of lipids in organism, and discover the connection between specific signaling pathways of lipid metabolism and disease (Lam and Shui, 2013). At present, mass spectrometry (MS) is the core tool for lipidomics, and liquid chromatography-mass spectrometry (LC-MS) and shotgun are mainstream in the MS method (Murphy and Nicolaou, 2013). The UPLC-Q-Orbitrap-HRMS method can extract the accurate mass of the target compound in the full scan mode, automatically trigger the acquisition of the tandem mass spectrum, reduce the probability of false positive results, and improve the accuracy of qualitative (Jiang et al., 2020). Therefore, a non-targeted lipidomics approach by using UPLC-Q-Orbitrap-HRMS was used for qualitative and semi-quantitative analysis of differential lipids.

In our work, H&E staining was performed and the histopathological changes were detected under microscope. The nontargeted lipidomics analysis of the serum of the mice was carried out to obtain the biomarkers of alcohol-induced liver damage, and to explore the mechanism of alcoholic liver damage and lipid metabolism of MFP against acute alcohol-induced liver damage.

## Materials and Methods

### Chemicals and Reagents

CH_2_Cl_2_, methanol, acetonitrile, isopropyl alcohol, and formic acid are HPLC grade from Sinopharm Chemical Reagent Co. (Shanghai, China). Ethanol, trinitrophenol, xylene, n-butanol, and formaldehyde are AR grade from FUYU Chemical Co. (Tianjin, China).

### Extraction, Purification and Characterization of *Mori Fructus* polysaccharides

Fresh MF was purchased from Beijing Tongrentang Co., Chengdu City, Sichuan Province, China. Fresh MF were pulverized, defatted, extracted by maceration in 90°C water and deproteinized to obtain an extract of MFP, then continued to precipitate with 30 and 50% ethanol to finally obtain crude polysaccharides (MFPA, MFPB). 20 ml of 6 mg mL^−1^ MFPA and MFPB solution was prepared and gradient eluted with 300 ml of distilled water and 300 ml of 0.05 mol L^−1^ NaCl on a DEAE-52 cellulose column (3 × 50 cm) at a flow rate of 2.5 ml min^−1^. Each eluate was monitored by the phenol-sulphuric acid method until no polysaccharide was present. MFPA1 and MFPB1 were obtained by combining the different fractions of MFP according to the absorption peaks, concentrating, dialyzing and freeze-drying under vacuum. The polysaccharide content, protein content, uronic acid content, molecular weight and monosaccharide composition of MFPA1 and MFPB1 were determined by sulfuric acid-phenol method, coomassie brilliant blue G-250 method, sulfuric acid-carbazole method, high performance gel chromatography and PMP pre-column derivatization high performance liquid chromatography, respectively. The results show that the contents of MFPA1 and MFPB1 were 93.44 and 89.68%, the specific experimental results are as described in the reference (Tan et al., 2021).

### Induction of Alcohol-Induced Liver Damage in Mice and Treatments

50 SPF-ICR mice (4–6 weeks, 18–22 g, males) were purchased from Changsha Tianqin Biotechnology Co., Ltd. All mice take food and drank water freely with standard environmental conditions. The mice were fed adaptively for a week and randomly divided into five groups, blank group, model group, bifendate group, MFPA1 group and MFPB1 group. At 10 o’clock every day, the blank group was given with saline (10 mg/kg), and other groups were administered intragastrically by 56% ethanol (10 ml/kg). At 12 o’clock every day, the bifendate group, MFPA1 group and MFPB1 group were administered by bifendate (220 mg/kg), MFPA1 (50 mg/kg), MFPB1 (50 mg/kg), and the blank group and model group were given with saline (10 mg/kg). After 7 day’s treatment, the blood and liver were collected for histopathological examination and nontargeted lipidomic analysis after the last administration.

### Histopathology Examination

The freshly dissected mice livers were cleaned, placed in a 10% neutral formalin solution for 36 h and stored at room temperature. The mice livers were removed, rinsed under running water for 5 min, cut into pieces, and dehydrated in increasing grades of ethanol, then cleared in xylene, finally embedded in paraffin. The paraffin blocks containing the liver tissue were cut into 4 µm thick pieces, stained with hematoxylin and eosin and the histological section continued to be sealed with neutral balsam and observed by optical microscope.

### Nontargeted Lipidomic Investigations

#### Extraction of Lipids in Serum

Our groups have adapted the Folch’s method, which is based on the theory of “the like dissolves like,” and successfully extracted lipids from serum samples (Folch et al., 1957). Briefly, whole blood of mice was collected in a 1.5 ml centrifuge tube and allowed to stand at room temperature for 30 min, followed by centrifugation at 3,000 rpm, 4°Cfor 10 min, and finally aspirated the upper layer of the yellow transparent serum in the centrifuge tube. 150 µL serum was placed in a 5 ml centrifuge tube and 600 μL of methanol, 300 μL of water and 450 μL of dichloromethane were added sequentially. The sample was mixed, homogenized, vortexed for 1 min and centrifuged at 12,000 rpm, 4°C for 15 min. The lower organic phase was taken and dried with nitrogen to obtain dried lipid extracts. The 200 μL of acetonitrile/isopropanol/water (65:30:5, V: V: V) mixture was added to dried lipid extracts, centrifuged at 8,000 rpm, 4°C for 5 min and the supernate was aspirated for nontargeted lipidomic analysis.

#### UPLC-Q-Orbitrap-HRMS Analysis

The serum nontargeted lipidomics method was used on a Q Exactive Focus quadrupole orbitrap high resolution mass spectrometry (Thermo Fisher Scientific, Shanghai, China) coupled with an Ultimate 3000 RSLC (HPG) ultra-performance liquid chromatography (Dionex, Beijing, China), with a HESI ionization source. The chromatographic parameters are shown in Table 1. All MS/MS data for serum lipids were collected in positive ion mode and negative ion mode by full scan mode. The optimized parameter settings for ESI were: spray voltage, 3.5 kV/3.2 kV; sheath gas, 35; auxiliary gas, 10 (both high-purity nitrogen); capillary temperature, 320°C; sphere-lens, 60 V; mass scan range, 100–1,000 m/z; resolution, 70,000; collision energy, 20,40 and 60 eV (Bian et al., 2021).To verify the stability of the instrument during operation, 20 μL from each serum lipid samples were taken, placed in the same tube and then evenly divided into five quality control (QC) samples. During the mass spectrometer analysis, one quality control sample was detected after every five serum lipid samples to test the stability of the experimental method.

**TABLE 1:** Chromatographic parameters for nontargeted lipidomics analysis.

	Chromatographic parameters
Column	Phenomenex ACE Excel 1.7 C18-AR UPLC PR column (100 mm × 2.1 mm, 1.7 μm)
Column temp	4°C
Injection volume	4 μL
Flow rate	0.3 ml/min
Run time	30 min
Mobile phase	A: 60:40 (v/v) acetonitrile/water containing 10 mM ammonium formate and 0.1% (v/v) formic acid
	B: 90:10 (v/v) isopropanol/acetonitrile containing 10 mM ammonium formate and 0.1% (v/v) formic acid
Gradient	0–2 min, 40%-43%B
	2–2.1 min, 43%-50%B
	2.1–12 min, 50%-54%B
	12–25 min, 54%-99%B
	25–25.1 min, 99%-40%B
	25.1–30 min, 40%B

### Statistical Analysis

The data obtained by statistical analysis are all expressed as mean ± SD. A one-way ANOVA was performed using Graphpad prism 8.0.2, with a significant difference between the two groups when *p* < 0.05. Pairwise comparison in five groups was conducted with HSD method. The raw data is processed by noise filtering, baseline correction and peak matching to obtain two-dimensional data consisting of m/z and peak area. After processing the missing values, the above data was standardized and conversion, and imported into SIMCA14.1 software for multivariate statistical analysis. Analysis of the results of the Principal component analysis (PCA) and Orthogonal partial least squares-discriminant analysis (OPLS-DA) showed differences in lipid metabolism between the groups. With VIP>1, *p* < 0.05, FC ≥ 1.5 or FC ≤ 0.67 as the screening conditions, the difference variables were obtained. By comparing the MS/MS spectrum of the difference variable with the standard MS/MS spectrum of the candidate lipid in the HMDB database, the differential lipid is finally obtained. The HMDB database IDs of candidate lipid were imported into MetaboAnalyst 5.0, and finally found signal pathways related to liver damage and MFP against liver damage.

## Results

### Effect of *Mori Fructus* polysaccharides on Alcohol-Induced Liver Damage

**FIGURE 1:**
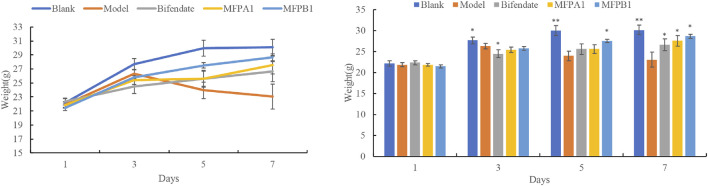
Body weight changes of mice, value are means ± SD (*n* = 10, g). *: *p* < 0.05, **:*p* < 0.01, blank, bifendate, MFPA1, MFPB1 group vs model group.

The body weight changes of the five groups of mice are shown in Figure 1. After a week of intragastric infusion with alcohol, the body weight of mice in the model group were significantly lower than those in other groups. After 3 days of intragastric infusion with alcohol, model mice began to lose weight, while the remaining four groups continued to gain weight until the end of the experiment, indicating that alcohol inhibited weight gain and bifendate, MFPA1 and MFPB1 were effective in improving this situation.

**FIGURE 2:**
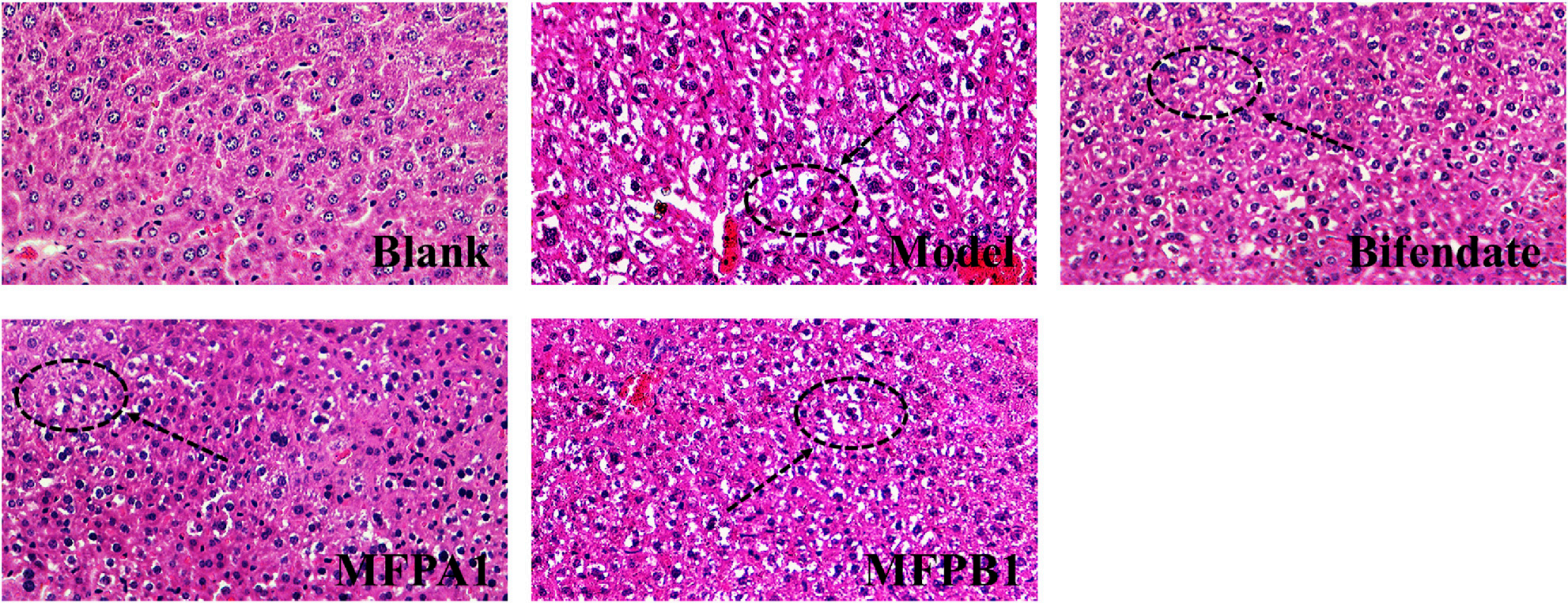
Histopathological changes of liver tissue in mice. Liver cells with lipid droplets are marked with black arrows.

The histopathological changes in the liver of the five groups of mice are shown in Figure 2. By using image pro plus 6.0 to count, the area ratios of hepatocyte ballooning degeneration of mice in the blank, model, bifendate, MFPA1 and MFPB1 groups were 1.95, 14.20, 4.76, 2.39 and 8.29% respectively. The liver cells of mice in the normal group were intact and regular, with orderly arrangement and obvious cell boundaries; the liver of mice in the model group showed inflammatory infiltration, ballooning degeneration in the cells and small lipid drops; the liver of mice in the bifendate, MFPA1 and MFPB1 groups showed similar symptoms to those of mice in the model group, but the lesions were less severe and the ballooning degeneration were reduced. The above results confirmed that MFP maybe attenuate alcohol-induced steatosis.

### Stability Detection of Experimental Method

**FIGURE 3:**
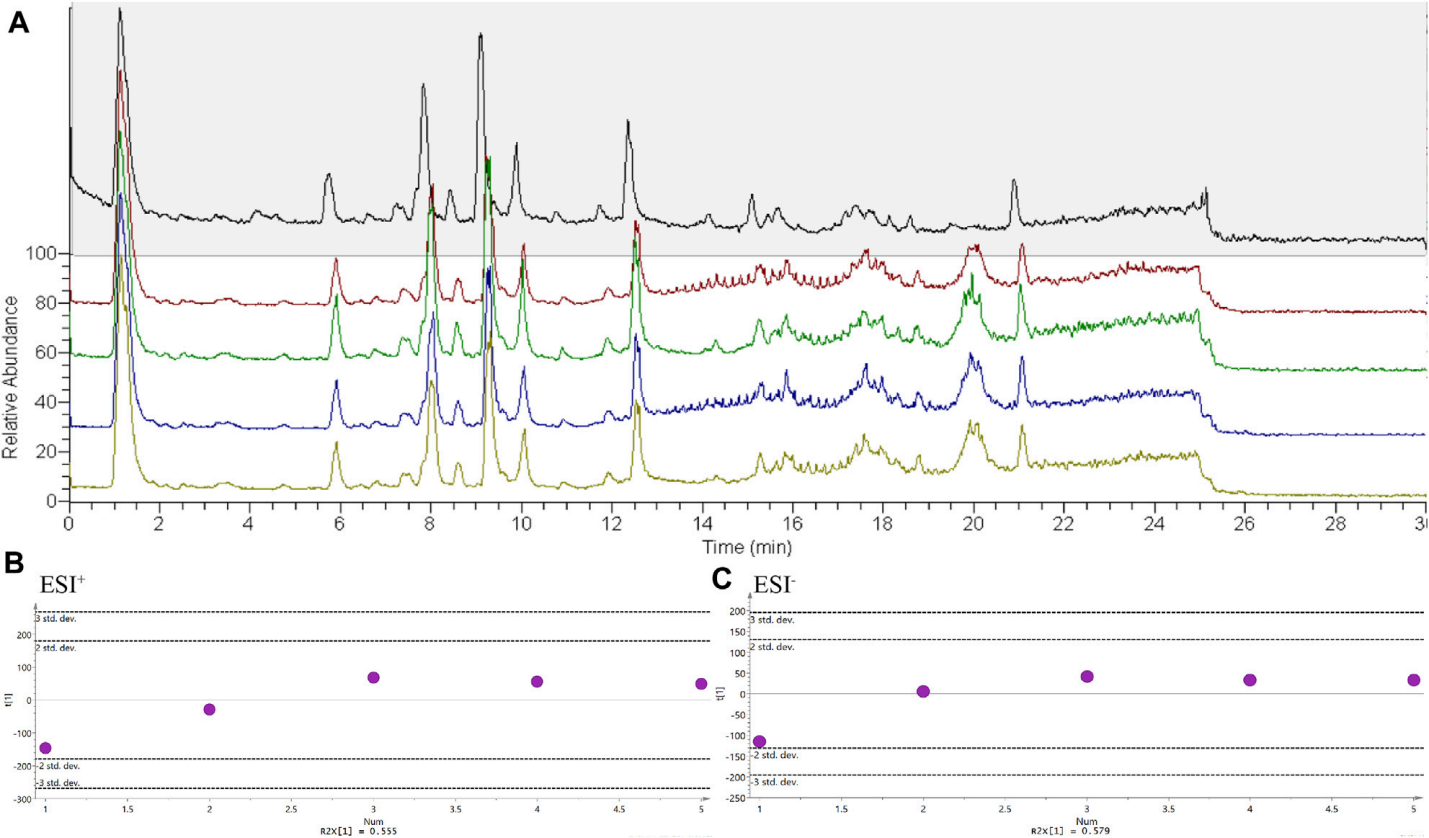
The stability detection of experimental method. *n* = 5. **(A)** Chromatograms of QC samples, **(B)** PCA score charts of QC samples in positive ion mode, **(C)** PCA score charts of QC samples in negative ion mode.

To test the stability of the mass spectrometry method, a QC sample was run every five serum lipid samples. The total ion chromatogram of the five QC samples is shown in Figure 3A. The number, time, and value of the chromatographic peaks are almost the same, indicating that the method was stable during lipid analysis. At the same time, PCA analysis found that the deviation of the five QC samples during operation was within 2std according to the results of lipid determination, which also confirmed that the mass spectrometry method was stable (Figures 3B,C).

### Multivariate Analysis of all Serum Lipids

Multivariate analysis of all lipids in the serum of five groups of mice can show the differences in lipid metabolism between groups and to obtain variable important to projection values (VIP) for all lipids in OPLS-DA.

**FIGURE 4:**
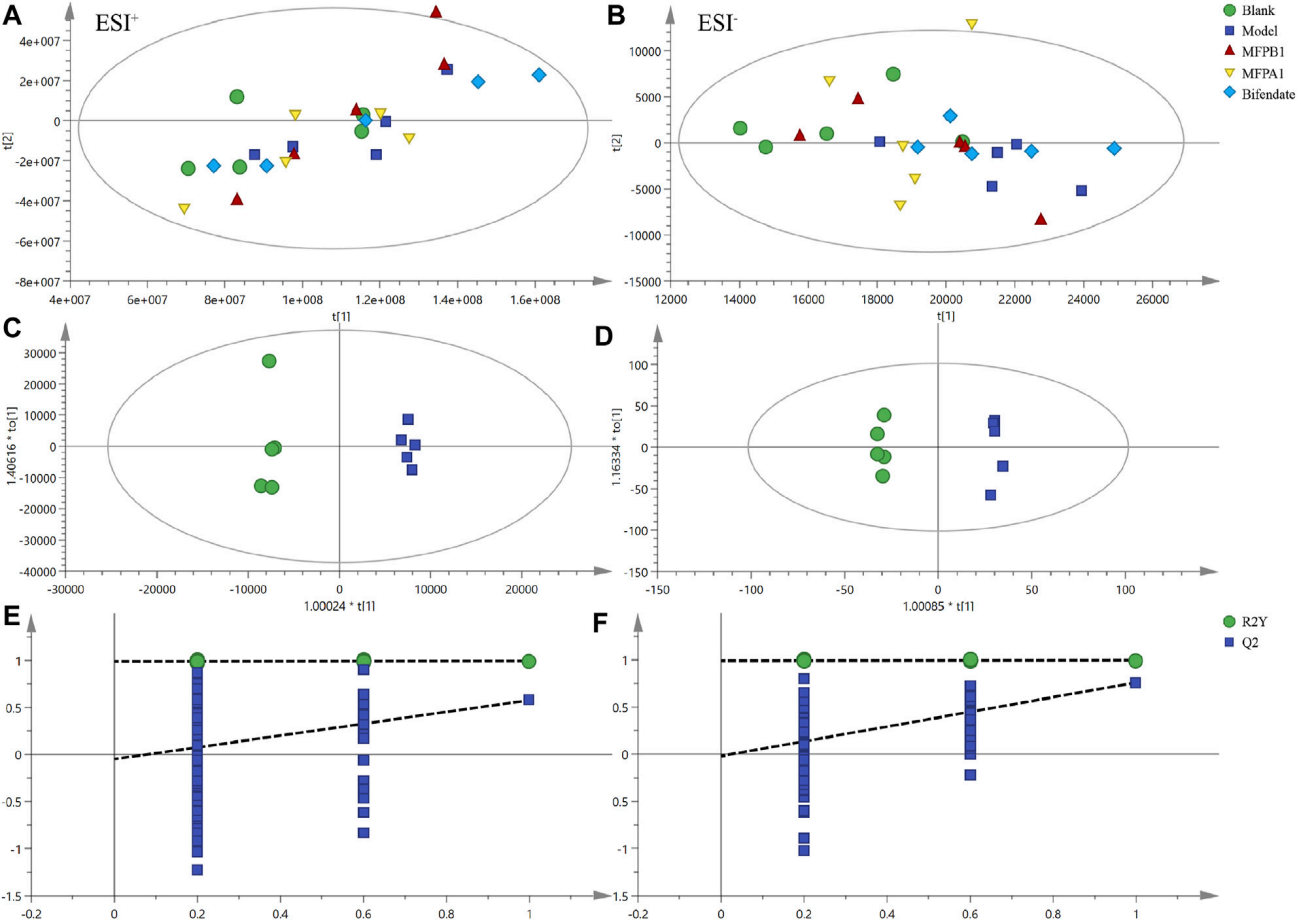
PCA score charts of serum lipid analysis of each experimental group are shown for positive ion mode **(A)** and negative ion mode **(B)**, OPLS-DA score charts of serum lipid analysis of each experimental group are shown for positive ion mode **(C)** and negative ion mode **(D)**, and permutation tests are shown for positive ion mode **(E)** and negative ion mode **(F)**. *n* = 5.

In the positive ion mode, PCA showed R2X = 0.994 and Q2 = 0.949, while OPLS-DA showed R2X = 0.893 and Q2 = 0.578. The result of 200 permutation tests confirmed that the OPLS-DA was not overfitted. The above results confirm that both models have good explanatory and predictive ability. As shown in Figures 4A,C,E, the mice in the model group were clearly separated from the mice in the blank group, implying that alcohol can cause disruption of lipid metabolism in mice serum, resulting in different lipid accumulation in the serum of the model and blank mice.

In the negative ion mode, PCA showed R2X = 0.958, Q2 = 0.837 and OPLS-DA showed R2X = 0.893, Q2 = 0.76. The result of 200 permutation tests confirmed that OPLS-DA did not overfit, indicating that both models had good explanatory and predictive ability. As shown in Figures 4B,D,F, there was a good separation between the blank group and the model group in the score of PCA and OPLS-DA, and the MFPA1 and MFPB1 groups were positioned closer to the blank group, implying that alcohol caused abnormal lipid accumulation in the serum of mice, and MFPA1 and MFPB1 maybe regulate the abnormal lipid accumulation and restore the levels of differential lipids to normal, resulting in the improvement of alcohol-induced liver damage.

### Screening and Identification of Differential Lipids

After data processing, the calculation of FC and one-way ANOVA were performed to finally obtain the P and FC values between the model and blank groups. With VIP>1, *p* < 0.05, FC ≥ 1.5 or FC ≤ 0.67 as the screening conditions, the difference variables were obtained. By comparing the MS/MS spectrum of the difference variable with the standard MS/MS spectrum of the candidate lipid in the HMDB database, seven kinds of differential lipids were precisely identified, including prostaglandins, long-chain fatty acids, glycerophospholipids, acyl carnitines. As shown in Table 2, the m/z of the measured molecular ions deviated from the m/z of the theoretical molecular ions within ±15 ppm; the concentrations of serum Palmitoylcarnitine, LPC(17:0), Palmitic acid, Vaccenic acid, Stearic acid, and Prostaglandin J2 in mice of the model group were lower than those in the blank group, while the concentration of LPC(18:3) in the serum of mice in the model group was higher than that in the blank group.

**TABLE 2:** Specific information of Palmitoylcarnitine, LPC (17:0), Palmitic acid, Vaccenic acid, Stearic acid, Prostaglandin J2 and LPC (18:3).

Metabolite	Species	ESI mode	Retention time (min)	Measured m/z	Mass accuracy (ppm)	MS/MS fragments	Blank	Model	Bifendate	MFPA1	MFPB1
Palmitoylcarnitine	CAR	+	11.76	400.3417	−1	85.0290; 400.3421	↑	—	—	↑	↑
Palmitic acid	FA	-	17.57	255.2328	2.35	205.7752;255.2332	↑	—	—	↑	↑
Vaccenic acid	FA	-	18.42	281.2486	−0.71	281.2489	↑	—	—	↑	↑
Stearic acid	FA	-	20.13	283.2641	1.77	283.2641	↑	—	—	—	—
Prostaglandin J2	PG	-	26.05	333.2072	0.3	271.2066;191.1437	↑	—	—	—	↓
LPC (18:3)	PC	-	6.46	562.315	0.89	277.2173;502.2940	↓	—	—	—	—
LPC (17:0)	PC	+	10.89	510.3553	−1.37	184.0733	↑	—	↑	↑	↑

Using the content of differential lipids in the model group as benchmark, and marked as "—". The up arrows (or down arrows) represented the relative up-regulation (or down-regulation) of the differential lipids, *p* < 0.05; “—” represented no change of the differential lipids content, *p* > 0.05. blank, bifendate, MFPA1, MFPB1 group vs model group. *n* = 5.

### Effect of Alcohol on Serum Lipid in Mice

**FIGURE 5:**
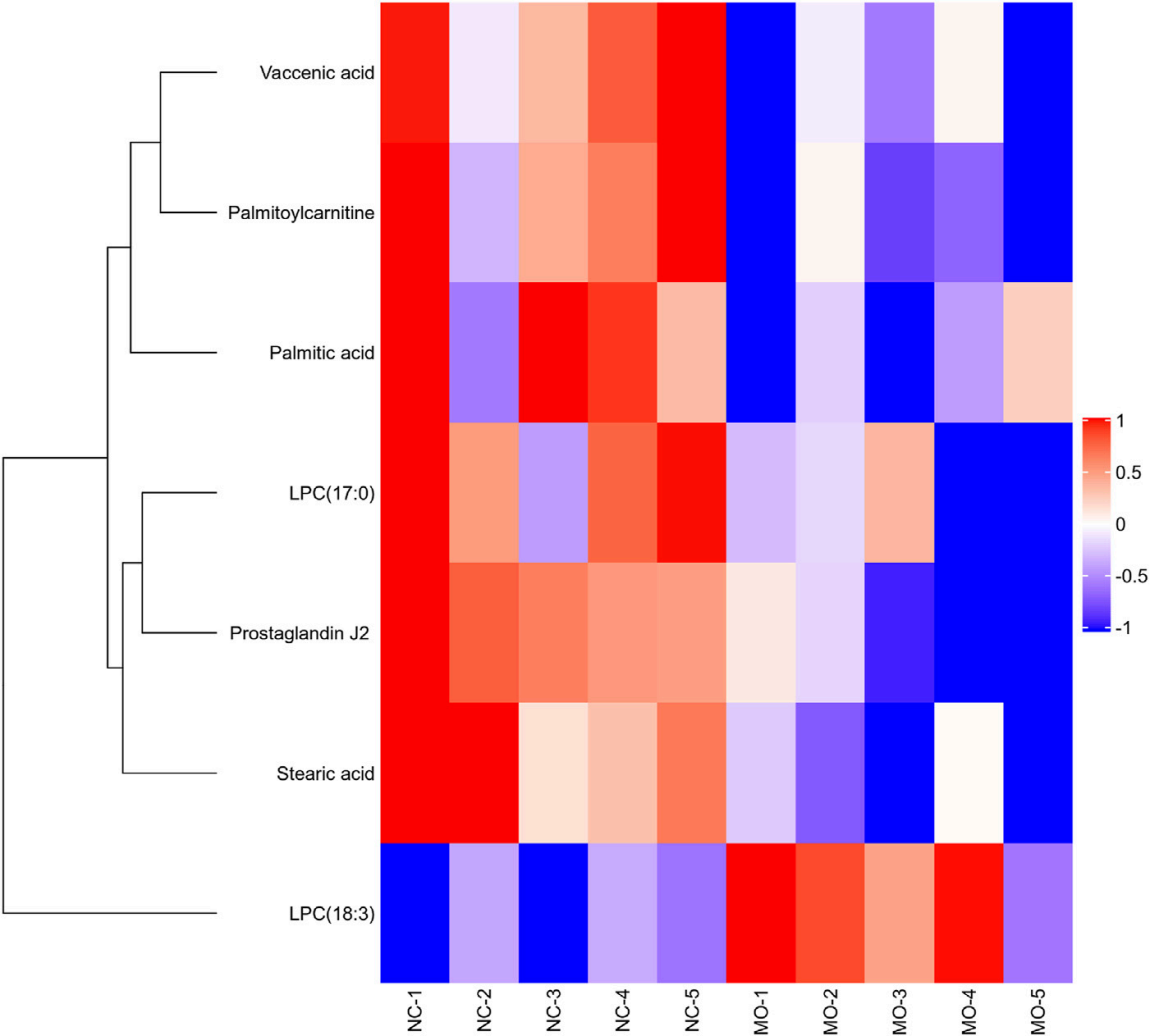
Overview of Matrix heatmap using fold-changes between groups.

**FIGURE 6:**
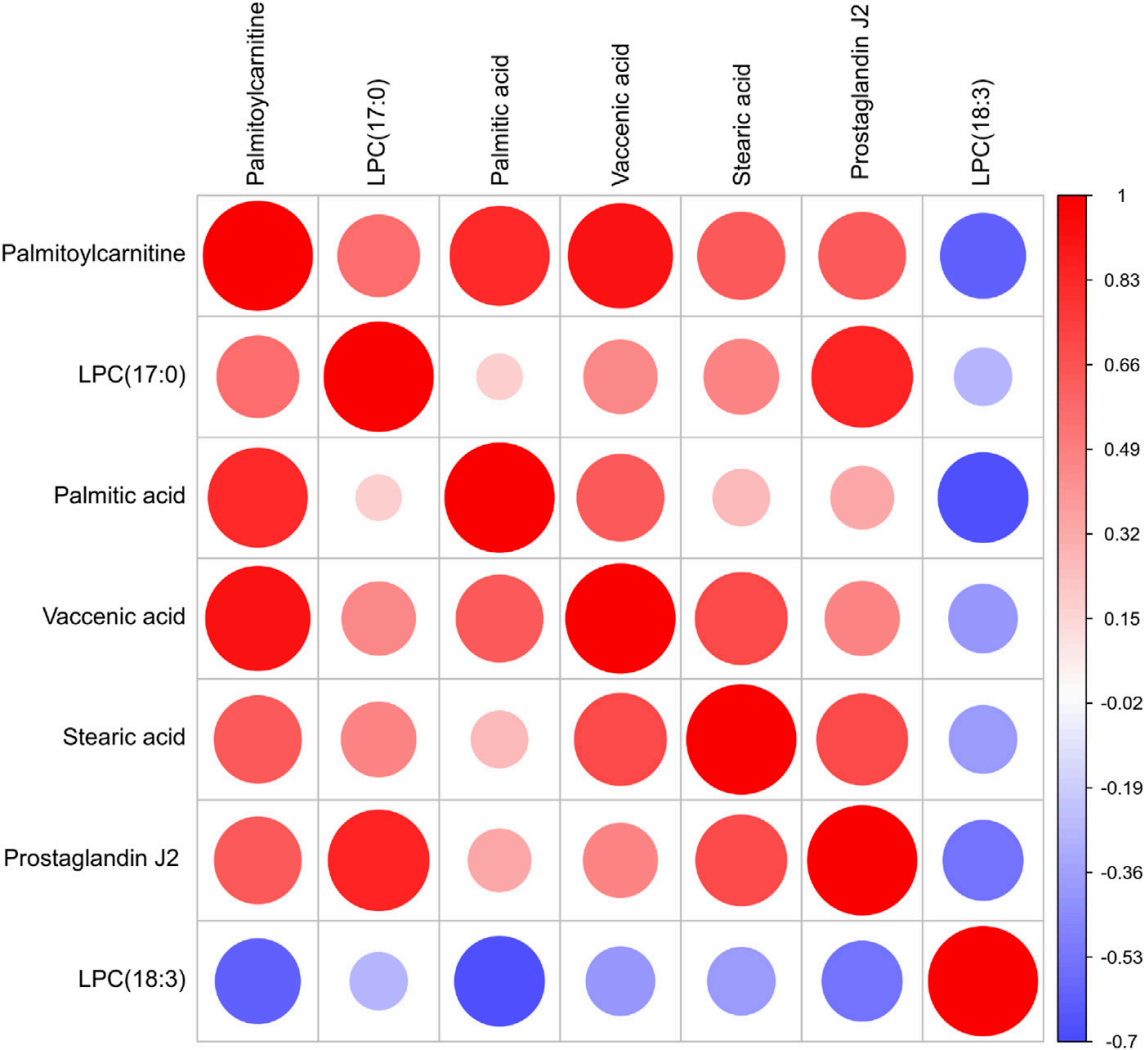
Correlation heat map of the seven differential lipids. The color and size of each circle represented the correlation value.

In our work, heatmap, correlation heatmap and bubble chart were presented based on the content of differential lipids and the ID of the HMDB database. As depicted in Figures 5, 6 the clustering algorithm classified the differential lipids into up-regulated and down-regulated lipids based on the results of changes in the content of the seven differential lipids, which showed that alcohol intake resulted in higher LPC(18:3) concentrations and lower Palmitoylcarnitine, LPC(17:0), Palmitic acid, Vaccenic acid, Stearic acid, and Prostaglandin J2 concentrations in mice serum. In order to explore the correlation between seven different lipids in alcohol-induced liver damage, the correlation analysis on seven different lipids in mice serum was performed. The results showed that Palmitoylcarnitine was significantly and positively correlated with Palmitic acid and Vaccenic acid, and Palmitic acid was significantly and negatively correlated with LPC(18:3), which implies that alcohol-induced Palmitoylcarnitine acted synergistically with Palmitic acid and Vaccenic acid during hepatic steatosis, while Palmitic acid acted antagonistically with LPC(18:3) during alcohol-induced liver damage.

**FIGURE 7:**
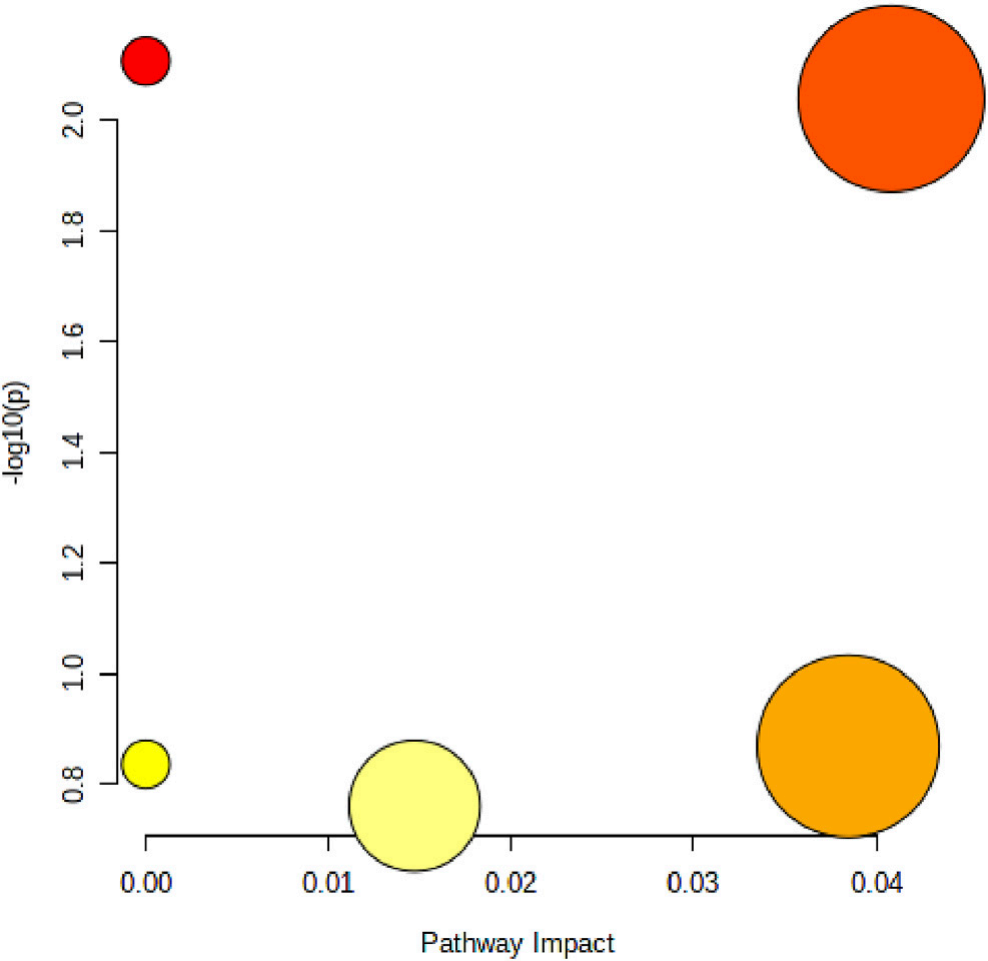
Disordered lipid metabolism pathway caused by alcohol. The size of the circle represents the pathway impact value (*X*-axis), and the lightness of the color represents the *p*-value weight (white to red, *Y*-axis).

In order to elucidate the mechanism of alcohol-induced liver damage, the HMDB database IDs of Palmitoylcarnitine, LPC (17:0), Palmitic acid, Vaccenic acid, Stearic acid, Prostaglandin J2 and LPC (18:3) were imported into MetaboAnalyst 5.0 that can explain the influence of alcohol on different lipid metabolism pathways. Present result indicated alcohol-induced liver damage may be related to the mechanism of fatty acid degradation, fatty acid biosynthesis, glycerophospholipid (Figure 7).

### Protective Effect of *Mori Fructus* polysaccharides in Mice With Alcohol-Induced Liver Damage

**FIGURE 8:**
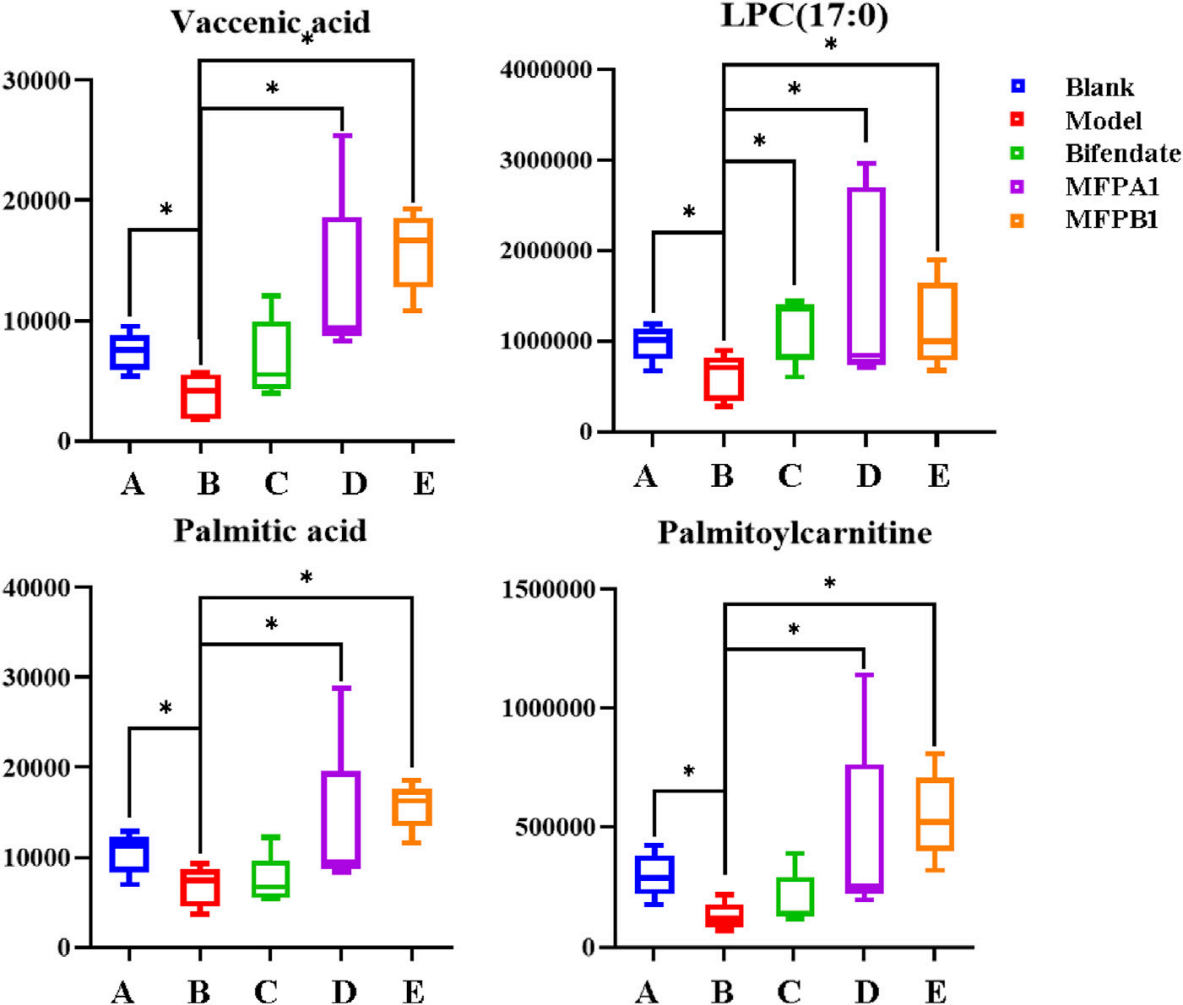
The content of the four differential lipids. *: *p* < 0.05, **:*p* < 0.01, blank, bifendate, MFPA1, MFPB1 group vs model group. *n* = 5.

The action mechanism of MFPA1 and MFPB1 against acute alcohol-induced liver damage was finally obtained by one-way ANOVA on the content of seven differential lipids among five groups of mice. As shown in Figure 8, the contents of Palmitoylcarnitine, LPC(17:0), Palmitic acid, and Vaccenic acid in the serum of mice decreased significantly after ingesting alcohol, but the contents of the above four lipids quickly returned to normal levels after 7 days of MFPA1 and MFPB1 administration. The statistical results showed that compared with the model group, the content of Palmitoylcarnitine in the serum of MFPA1 group and MFPB1 mice increased by 260.26 and 345.05%, respectively; the content of LPC(17:0) increased by 154.18 and 93.26%, respectively; the content of Palmitic acid increased by 94.51 and 131.55%, respectively; the content of Vaccenic acid increased by 239.32 and 320.81%, respectively.

**FIGURE 9:**
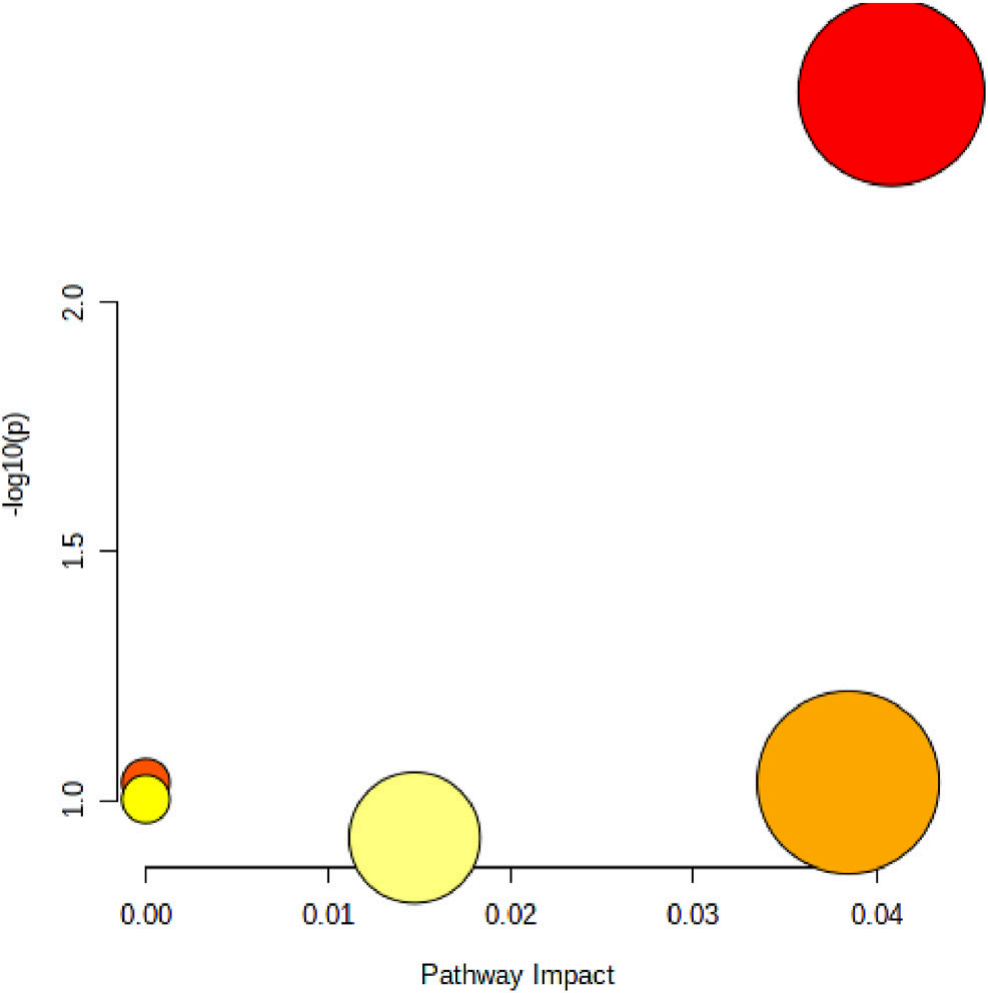
Abnormal lipid metabolism pathway that MFP may be attenuate. The size of the circle represents the pathway impact value (*X*-axis), and the lightness of the color represents the *p*-value weight (white to red, *Y*-axis).

Pathway analysis of almitoylcarnitine, LPC (17:0), Palmitic acid, and Vaccenic acid showed that MFPA1 and MFPB1 may regulate fatty acid degradation, fatty acid biosynthesis, and glycerophospholipid metabolism to reduce alcohol-induced liver damage (Figure 9).

## Discussion

Studies have shown that saturated fatty acids have a therapeutic effect on alcohol-induced liver damage, and Palmitic acid is one of the most common saturated fatty acids found in animals (Sveinbjrnsson and Baldursdóttir, 2020). Chronic alcohol consumption increases the levels of sterol regulatory element binding protein-1 (SREBP-1) and Acetyl CoA carboxylase (ACC), leading to the accumulation of triacylglycerols in the liver, while saturated fatty acids inhibit the activity of SREBP-1 and also increase the transport ability of triacylglycerols, thereby reducing the liver damage caused by alcohol consumption (Cheng and Kaplowitz, 2003; You, 2002; Park et al., 2002). Saturated fatty acids can reduce liver lipid peroxidation by affecting cytochrome P450 2E1 and non-heme iron, and reduce the expression of inflammatory factors and cytokines such as Cyclooxygenase-2 and TNF-α by down-regulating NF-κB (Ming et al., 1999; Lawler et al., 1998). At the same time, the concentration of lysophosphatidylcholine containing polyunsaturated fatty acids in the serum of alcohol-induced liver damage mice is higher than that of normal mice, while the content of saturated lysophosphatidylcholine is lower than that of normal mice (Li et al., 2011).Alcohol intake increases the liver’s uptake of exogenous palmitic acid and subsequently incorporates palmitic acid into the liver’s triglycerides or total lipids. The upregulation of fatty acid transporters (FATP), especially CD36/FAT, FATP1 and FATP5, promotes fatty acid uptake, fat accumulation and steatosis in mice (Nie et al., 2020). At the same time, both SREBP-1 and ChREBP may be activated under alcohol intake, which further promotes the production of fatty acids.

### Effect on Fatty Acid Degradation Metabolism

Palmitic acid is one of the most common fatty acids in animals, its concentration in serum is related to synthesis and degradation. As a long-chain fatty acid, Palmitic acid is activated by the catalysis of CoA synthase to become fatty acyl CoA, which is transported by carnitine to the mitochondria, where it is finally β-oxidised to produce acetyl CoA (Fromenty and Pessayre, 1994; Hu et al., 2016). Palmitoylcarnitine is an acylcarnitine, formed from carnitine transported by the carnitine transporter, which facilitates the transfer of long-chain fatty acids from the cytoplasm to the mitochondria (Serviddio et al., 2011). In our work, the levels of Palmitic acid and Palmitoylcarnitine in the alcohol-induced liver damage mice decreased, while the levels of Palmitic acid and Palmitoylcarnitine in the mice of the MFPA1 and MFPB1 groups increased, which means that both alcohol and MFP may affect the degradation of fatty acids by regulating the concentration of palmitic acid and palmitoylcarnitine.

### Effect on Glycerophospholipid Metabolism

Lysophosphatidylcholine (LPC), which accounts for approximately 8–12% of plasma, is a class of compounds produced by the hydrolysis of phospholipids and the loss of one molecule of fatty acid. The synthesis and catabolism of LPC takes place mainly in the liver, and its concentration in plasma fluctuated greatly. Numerous liver diseases have been found to cause changes in plasma LPC levels, making LPC a potential biomarker for liver disease (Takahashi et al., 2002; Tanaka et al., 2012). In this study, the level of LPC(18:3) was increased and the level of LPC(17:0) was decreased in mice of the model group, while the level of LPC(18:3) was decreased and the level of LPC(17:0) was increased in mice of the MFPA1 and MFPB1 groups. This result is consistent with a previous study reporting an increased polyunsaturated LPC and decreased saturated LPC measured by metabolomics study in alcoholic fatty liver mice (Li et al., 2011). Meanwhile, MFP may affect glycerophospholipid metabolism by regulating the concentration of LPC (17:0).

### Effect on Fatty Acid Biosynthesis Metabolism

Palmitic acid is the first fatty acid produced during fatty acid synthesis, as well as being a precursor to many long-chain fatty acids. Acetyl CoA in the mitochondria crosses the mitochondrial membrane into the cytoplasm, where Palmitic acid is generated under the combined effect of acetyl CoA carboxylase and fatty acid synthase (Foster., 2012). In our study, the serum Palmitic acid level of model group mice decreased, indicating that alcohol affected the synthesis of Palmitic acid. MFP could restore the level of Palmitic acid to normal, indicating that MFP promoted the synthesis of citric acid.

## Conclusion

In this work, histopathological examination of the livers of mice with acute alcohol--induced liver damage showed that alcohol caused lipid droplets to appear in the liver cells, leading to steatosis and ballooning degeneration. Compared with the model group, the liver cell structure of the MFP group was more complete and the cytoplasm was more evenly distributed. These results suggest that the model was successfully induced and that MFP had a protective effect on the liver. Nontargeted lipidomic analysis have shown that alcohol causes disruption of lipid metabolism in serum, and alcohol intake and MFP intervention have significant effects on fatty acid synthesis, degradation and glycerophospholipid metabolism.

## Data Availability

The original contributions presented in the study are included in the article/Supplementary Material, further inquiries can be directed to the corresponding author.
